# Varicella Zoster Virus Meningitis in a Young Immunocompetent Adult without Rash: A Misleading Clinical Presentation

**DOI:** 10.1155/2014/686218

**Published:** 2014-12-29

**Authors:** Thomas Pasedag, Karin Weissenborn, Ulrich Wurster, Tina Ganzenmueller, Martin Stangel, Thomas Skripuletz

**Affiliations:** ^1^Department of Neurology, Hannover Medical School, Carl-Neuberg-Straße 1, 30625 Hannover, Germany; ^2^Department of Psychiatry and Psychotherapy, Klinikum Region Hannover, Rohdehof 3, Langenhagen, Germany; ^3^Institute of Virology, Hannover Medical School, Carl-Neuberg-Straße 1, 30625 Hannover, Germany

## Abstract

Meningitis caused by varicella zoster virus (VZV) is rare in healthy population. Predominantly immunocompromised patients are affected by reactivation of this virus with primary clinical features of rash and neurological symptoms. Here we report a young otherwise healthy man diagnosed with a VZV meningitis without rash. He complained of acute headache, nausea, and vomiting. The clinical examination did not show any neurological deficits or rash. Cerebrospinal fluid (CSF) analysis revealed a high leukocyte cell count of 1720 cells/*µ*L and an elevated total protein of 1460 mg/L misleadingly indicating a bacterial infection. Further CSF analyses, including polymerase chain reaction (PCR) and detection of intrathecal synthesis of antibodies, showed a VZV infection. Clinical and CSF follow-up examinations proved the successful antiviral treatment. In conclusion, even young immunocompetent patients without rash might present with VZV meningitis. CSF examination is a key procedure in the diagnosis of CNS infections but in rare cases the standard values cell count and total protein might misleadingly indicate a bacterial infection. Thus, virological analyses should be considered even when a bacterial infection is suspected.

## 1. Introduction

Infections with neurotropic herpes viruses (herpes simplex type 1/2, varicella zoster virus (VZV)) are frequent in humans. These viruses persist within cranial nerves, dorsal roots, and autonomic ganglia causing latent infections with the ability of reactivation [1–3]. Reactivation of VZV shows mainly a herpes zoster presenting with rash and pain affecting the entire dermatome and less frequently a zoster sine herpete [1–3]. VZV infection of the central nervous system (CNS) such as encephalitis, meningitis, myelitis, or angiitis occurs less frequently but is feared because of the numerous unfavourable outcomes [1, 3, 4]. Usually CNS infection with VZV comes along with dermal affection but can rarely develop without rash [1, 3, 5–10].

Acute infection or VZV reactivation affects predominantly older individuals and/or immunocompromised patients [1–3, 9]. CNS infection with VZV in young healthy adults is rare and is unexpected and only very few cases have been described so far [5–9]. Here, we describe a young previously healthy man with VZV meningitis who had only minimal symptoms.

## 2. Case Presentation

An 18-year-old man experienced severe occipital headache accompanied by nausea and vomiting. All symptoms started immediately after a backward roll doing judo exercise. Treatment with paracetamol, acetylsalicylic acid, and metamizole did not show beneficial effects. Thus, he presented to our emergency room nine days after the first symptoms.

The physical examination was normal. Particularly, stiff neck as a typical sign of meningitis was not found and he did not show any rash. He had never been sick before and did not take any medications regularly. Furthermore, he did not smoke or drink alcohol excessively.

Subarachnoid hemorrhage or dissection of cerebral arteries was first considered. Magnetic resonance imaging (MRI) scan of the brain showed no abnormalities such as bleeding, infarction, or malignancy. Additional MR-angiography and ultrasound examination of carotid, vertebral, and intracranial arteries revealed no vascular alterations. Measurements of body temperature and blood pressure as well as laboratory urine and blood examinations (blood count, sodium, potassium, C-reactive protein, creatinine, transaminases, creatinine kinase, serum protein electrophoresis, thyroid-stimulating hormone, thyroxine, triiodothyronine, anti-thyroid autoantibodies (anti-thyroid peroxidase antibodies, thyrotropin receptor antibodies, and thyroglobulin antibodies), anti-nuclear antibodies, anti-neutrophil cytoplasmic antibodies, anti-cardiolipin antibodies, angiotensin-converting enzyme, and amount of vitamins B12, B6, and B1) showed normal results.

CSF analysis revealed a high leukocyte cell count of 1720 cells/*μ*L and an elevated total CSF protein of 1460 mg/L. Furthermore, slightly elevated CSF lactate concentration of 3.0 mmol/L and a slightly reduced CSF glucose concentration of 42 mg/dL were found. Considering a bacterial infection, intravenous treatment with ceftriaxone (2 g/day) and ampicillin (15 g/day) was applied.

Cytologic analysis of the CSF revealed 3% plasma cells. The remaining cells were predominantly lymphocytes with normal morphology. Further CSF abnormalities included elevated albumin quotient (QAlb) indicating a moderate blood-CSF-barrier-dysfunction (Table 1). Intrathecal immunoglobulin (Ig) synthesis of IgG, IgM, and IgA as calculated based on the method of Reiber-Felgenhauer [11] was not found. Oligoclonal bands restricted to the CSF were identified indicating intrathecal IgG synthesis in the CSF. Furthermore, identical oligoclonal IgG bands in CSF and serum were found indicating a systemic humoral immune response towards foreign antigens and/or self-antigens.

Microbiological analyses did not detect any bacterial infection. Further analyses for* Borrelia burgdorferi*,* Treponema pallidum*,* Mycobacterium tuberculosis*, toxoplasmosis,* Candida*, and* Cryptococcus neoformans* showed negative results. Virological examinations presented negative results for herpes simplex virus, cytomegalovirus, Epstein-Barr virus, enteroviruses, tick-borne encephalitis, human immunodeficiency virus, and hepatitides B, C, and A. Quantitative real-time PCR analysis [12] of the CSF revealed presence of VZV-DNA with a concentration of 50.000 copies/mL indicating high viral replication. Thus, one day after antibiotic therapy the patient was treated with acyclovir (2250 mg/day intravenously for 11 days), followed by valacyclovir (3000 mg/day orally for 5 days). Additionally, we measured the intrathecal synthesis [13] of VZV immunoglobulin G antibodies (Enzygnost Anti-VZV/IgG, Siemens Healthcare Diagnostics) and found a specific antibody index (AI) of 74.9 (normal value < 1.5). The presence of a strong intrathecal IgG production against VZV confirmed the VZV infection in the CNS.

Eleven days after intravenous therapy, the patient was discharged feeling well. Follow-up CSF examination was performed 23 days after the end of antiviral treatment. A slight pleocytosis with 19 cells/*μ*L was still found. Plasma cells were not found and lymphocytes and monocytes showed normal morphology. VZV-PCR was negative but a persisting intrathecal IgG production to VZV (specific antibody index of 21.5) indicated a preceding VZV infection. The patient felt well without any symptoms. He practiced judo again four times per week.

## 3. Discussion

Here we present a young previously healthy man with a VZV meningitis without rash. This case is extraordinary because the clinical presentation was unusual for a patient with meningitis and the initial CSF findings with very high pleocytosis and elevated total CSF protein initially misleadingly suggested a bacterial infection. Interestingly, further CSF examinations detected a VZV infection.

Our case underlines the importance of specialised CSF diagnostics in acute neurological emergency situations. CSF examination is generally considered a key procedure in the diagnosis of CNS infections [14]. Using sensitive laboratory analyses (e.g., PCR and detection of intrathecal production of specific antibodies) recent epidemiological studies found a portion of 5–29% of VZV in aseptic meningitis and encephalitis and it was suspected that VZV infections had been underestimated in earlier publications [9, 15–18]. Nevertheless in immunocompetent patients without rash and neurological deficits (as in our case) VZV meningitis seems to be rare and only few cases have been described to date (see Table 1). Infections of the CNS are accompanied by an elevated cell count in the CSF. In large series including patients with aseptic meningitis and encephalitis CSF findings predominantly revealed lymphomonocytic pleocytosis of less than 500 cells/*μ*L, mild to moderately elevated total protein, and normal lactate levels [16–18]. In patients with VZV infection median cell counts of 43/*μ*L, 132/*μ*L, 286/*μ*L, and 293/*μ*L were found and the cell counts ranged from 15 to 840 cells/*μ*L [9, 16–18]. In our case, we found the highest pleocytosis (1720 cells/*μ*L) that has been described for this group of patients. In addition, total protein and lactate concentration were elevated leading to a misleading diagnosis of bacterial meningitis.

In conclusion, even young and previously healthy patients without clinical features of dermal irritation such as rash might present with VZV meningitis. We highlight the importance of considering VZV as a possible cause for meningitis even in previously healthy young patients and the recommended diagnostic lumbar puncture. Detailed CSF diagnostic procedures including PCR and detection of intrathecal synthesis of antiviral antibodies (especially for VZV and HSV) should be considered even though CSF cell count and total protein seem to indicate a bacterial infection.

## Figures and Tables

**Table 1:** Clinical and CSF findings in immunocompetent patients with VZV meningitis without rash. Qalb: albumin quotient (CSF albumin/serum albumin), OCB: oligoclonal bands, and ASI: antibody specific index ((CSF VZV-IgG/serum VZV-IgG)/(CSF total-IgG/serum total-IgG)). MRI results of patient 4: MRI revealed an ill-defined T2 hyperintensity in the right frontal lobe, extending from the cortical surface to the frontal horn of the lateral ventricle without mass effect, consistent with a hamartoma or cortical dysplasia. A follow-up MRI with gadolinium on day 10 was normal, with no change in the solitary T2 hyperintensity.

Patients	Age/ gender	Symptoms	Duration of symptoms (days)	Clinical signs	Previous diseases	Brain imaging	Cerebrospinal fluid								Reference
							Cells/μL	Cells (%)	Protein (mg/L)	Lactate (mmol/L)	Qalb	OCB	VZV DNA	VZV ASI	
1	18/male	Headache, nausea, vomiting	9	Examination normal, no rash	None	MRI: normal	1720	96% lymphocytes, 3% plasma cells, 1% monocytes	1460	3.0	19.7	Type 3	50 000 copies/mL	74.9	Our case
		No symptoms	48	Examination normal, no rash	None	n.d.	19	95% lymphocytes, 5% monocytes	613	1.6	8.1	Type 3	Negative	21.5	Our case

2	53/female	Headache, nausea, photophobia, generalized body aches	3	Examination normal, no rash	Diabetes mellitus, bronchial asthma, bipolar disease	CT: mild atrophy	923	98% lymphocytes, 2% monocytes	1650	3.1	n.d.	n.d.	Positive	n.d.	Klein et al., 2010 [8]

3	12/male	Headache	4	Examination normal, no rash	Bronchial asthma	CT: normal	590	97% lymphocytes, 1% monocytes, 2% neutrophils	860	n.d.	n.d.	n.d.	Positive	n.d.	Jhaveri et al., 2003 [5]

4	26/female	Headache, nausea, photophobia, vomiting	6	Examination normal, no rash	None	MRI (see legend)	331	99% mononuclear	2190	n.d.	n.d.	n.d.	127 000 copies/mL	n.d.	Habib et al. 2009 [7]

5	14/male	Headache, photophobia, vomiting	7	Examination normal, no rash	Migraine	MRI: normal	285	n.d.	1580	n.d.	n.d.	n.d.	Positive	n.d.	Leahy et al., 2008 [6]
